# An Unusual Appearance of Base of First Metacarpal Fracture: A Case Report

**DOI:** 10.7759/cureus.24353

**Published:** 2022-04-21

**Authors:** Sebastian Kosasih, Andreas Georgiou, Norbert V Kang

**Affiliations:** 1 Department of Plastic and Reconstructive Surgery, Royal Free Hospital, London, GBR

**Keywords:** trapeziectomy, carpometacarpal subluxation, rolando fracture, bennett’s fracture, base of first metacarpal fracture, first metacarpal fracture

## Abstract

We present the case of an unusual base of the first metacarpal fracture. The presentation and radiological images are provided to demonstrate a first metacarpal base fracture but with a concurrent appearance at first glance of a trapeziectomy mimic on plain radiographs. The CT scan however demonstrates the true nature of the injury - a comminuted fracture with carpometacarpal subluxation. The radiological and clinical findings presented a diagnostic and therapeutic dilemma. We elected to not intervene surgically with a good resulting clinical outcome, reminding us of the need to treat the patient and not their radiographic images.

## Introduction

Fractures of the base of the first metacarpal are common and occur after axial overloading along the shaft causing compression of the first carpometacarpal joint (CMCJ). As in other fractures, they can be classified as extra or intra-articular, with common fracture patterns of the latter including Bennett’s and Rolando fractures, and comminuted fractures often being treated as a severe form of Rolando fracture [1]. Thumb metacarpal fractures are more tolerant of rotation and angulation than other fingers due to compensation from the adjacent joints [2]. However, they are generally unstable and are often managed operatively, though this remains controversial. Here we present a case of a misleading plain radiograph of a base of thumb comminuted fracture, with the full extent of injury only fully appreciated on a CT scan. This case proved a diagnostic and therapeutic dilemma and was eventually managed non-operatively with good functional outcomes. The patient has given informed consent for this report.

## Case presentation

A 64-year-old male patient fell from his bicycle onto his right hand, resulting in an axial loading force and hyperabduction of the thumb. The patient is right-hand dominant with no underlying comorbidities and presented to our clinic three weeks later. He only presented because of continuing but intermittent pain in his thumb though he was functionally well preserved. His main concern was the possibility of a disruption to his clerical duties, and his hobby of painting if the pain did not resolve completely. On examination, there was minimal swelling of the hand, no tenderness, and an unrestricted range of motion of the thumb.

**Figure 1:**
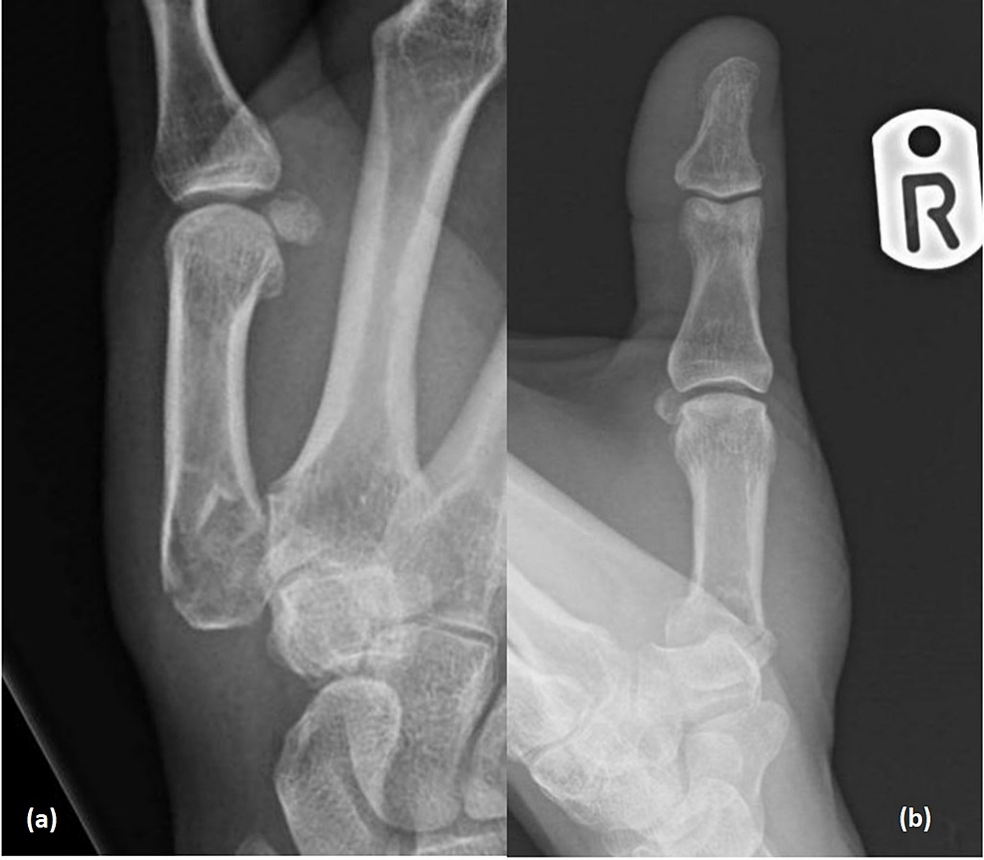
Plain x-rays of right thumb showing fracture at base of first metacarpal with no clear appearance of trapezium. (a) Lateral view of first metacarpal base fracture with minimal comminution and appearance of trapeziectomy. (b) Antero-posterior view of first metacarpal base fracture with possible intra-articular element but no obvious comminution.

**Figure 2:**
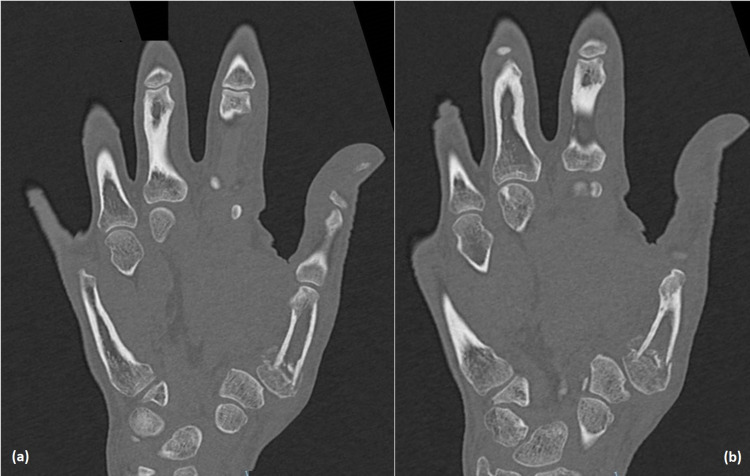
CT scan of right hand showing comminuted fracture of the base of the first metacarpal and the trapezio-metacarpal joint. Slices of CT scan in coronal view demonstrating intra-articular fracture pattern in (a), and carpo-metacarpal joint subluxation with multiple extruded small metacarpal fragments on the ulnar side not visible on plain films in (a) and (b).

Plain x-rays (Figures 1a, 1b) showed a mildly comminuted fracture of the base of the first metacarpal, with a somewhat obscured view of the trapezium on the lateral and resulting apparent loss of the trapezio-metacarpal articulation. Whilst at first glance we initially thought the patient had undergone a prior trapeziectomy, the trapezium was more visible on the anteroposterior view. However, given the degree of subluxation or possible trapezial injury required to create this appearance, we felt that a CT scan would clarify the overall injury pattern. This subsequently confirmed the true nature of the metacarpal fracture and the arrangement of the carpal bones (Figures 2a, 2b).

Due to the relative lack of pain and good function, we elected not to intervene surgically. After non-operative management was decided (at week 3 post injury due to his delayed presentation), the hand therapy team placed him into a fibreglass cast to wear full time for four weeks until tenderness on palpation had resolved (immobilising thumb carpometacarpal and metacarpophalangeal joints but leaving the interphalangeal joint and other fingers free). At week 7 post injury, he was converted into an off the shelf neoprene splint with thumb spica extension for protection and comfort, and an active range of motion protocol of the wrist and thumb was started. Gradual strengthening exercises were then started from week 10 post injury. At 20 weeks post injury, full active range of motion was objectively reported by the hand therapy team, and the patient reported that he was back to full function without limitations including doing yoga and cycling without pain at which point he was discharged from follow up with advice to return if problems develop.

## Discussion

We have presented a case of a middle-aged right-hand dominant gentleman presenting to our hand trauma clinic three weeks after an axial loading and hyperabduction injury to the right thumb, with a radiograph appearing to show a loss of the trapezium in addition to a base of first metacarpal fracture. What makes this case unusual is the differing appearances of the initial radiographs and the later CT scan - an apparently minimally comminuted base of metacarpal fracture with loss of trapezium on plain films, with the true injury only being clarified by a CT scan showing a high degree of comminution at the base of the first metacarpal including an intra-articular element and a subluxed but present CMCJ. The relative absence of fracture fragments on the plain x-rays and the excellent range of motion suggested that the injury was relatively minor. It is only the CT scan that shows the true extent of the injury. Despite the high level of comminution at the fracture site on CT suggesting a high force trauma, after extensive discussion with the patient and considering the good level of function, we elected to manage this case non-operatively with good results at the time of discharge from hand therapy at five months.

Generally, the base of first metacarpal fractures is managed slightly differently than other metacarpal base fractures - a lack of interossei and the transverse metacarpal ligament gives less surrounding stability [3]. The deformity can often result due to the pull of abductor pollicis longus (APL), extensor pollicis longus (EPL), and adductor pollicis (AP) resulting in fractures that can deform with the distal fragment in an apex dorsal, adducted and flexed position [4]. Surrounding joint compensation and increased mobility mean that the thumb is more tolerant of rotation and angulation than other fingers; however, despite this, there is still a role for operative management. Apex dorsal angulation of more than 30 degrees is usually an indication for operative management as they often do badly functionally, with narrowing of the first webspace being problematic due to hyperextension of the metacarpophalangeal joint which is poorly tolerated [1].

Regarding the literature base on operative versus non-operative management for first metacarpal base fractures, until the 1980s there was some evidence of reasonable outcomes with closed reduction and thumb spica casting or splinting for intra-articular fractures [5]. More recent studies, however, have disputed this idea but there is still controversy in the field. Despite this, operative management of intra-articular fractures is increasingly common, particularly as issues with soft tissue rehabilitation must also be considered with prolonged casting [6-8]. Indications for operative management (closed reduction and K wiring, open reduction and internal fixation with plates or screws, or external fixation) include extra-articular fractures with over 30 degrees of angulation post-reduction; loss of reduction following non-operative management; and intra-articular fractures - Bennett’s fractures over 1mm displaced or any Rolando fracture (with comminuted fractures usually being treated as Rolando fractures [1]). In this case, whilst our patient has a fracture pattern that would usually be amenable to an operation, after discussion with the patient and looking at his level of function we elected to manage this injury non-operatively given the good range of motion and minimal tenderness at three weeks post injury (the time of presentation). He underwent splinting and hand therapy, and the patient-reported satisfaction with his outcome and experience has been high at the time of discharge from hand therapy at five months. This case of a comminuted base of first metacarpal fracture meeting indications for an operation but being managed non-operatively is evidence that despite objectively meeting radiological criteria for operative management, the overall picture must be considered, and non-operative treatment is still a valid and viable treatment strategy in selected patients.

Whilst it has been noted that five months is still an early outcome and post-traumatic arthritis is possible, no operative management is without its own risks, and arthritis (amongst other problems) is still a recognised complication even with gold standard operative management. As the patient had a full return to normal active range of motion at the time of discharge from hand therapy, even if post-traumatic arthritis occurs further down the line surgical management remains an option and may in fact be easier than if his hand had been operated on with this injury due to a lack of post-surgical scarring.

With respect to the initial imaging, the cause of the radiograph appearance is not entirely clear. First carpometacarpal joint subluxation is a recognised feature of osteoarthritis (OA) due to laxity in the joint capsule, forming part of the Eaton-Littler radiological criteria for grading the base of thumb OA [9]. However, given the patient’s relatively acute pain presentation it is unlikely that subluxation related to OA alone would have been severe enough to account for the appearance of trapeziectomy on plain radiographs, and there is little other evidence of OA in the radiographs. For this reason, we felt that cross-sectional imaging was a useful adjunct in this scenario to elucidate the true nature of the radiographic appearance. With the pattern on CT showing subluxation of the first carpo-metacarpal joint and associated comminuted base of first metacarpal fracture, an alternative more plausible explanation therefore would be that the hyperabduction and axial loading force almost fully dislocated the metacarpo-trapezial joint, stopped only by the base of the first metacarpal fracturing in place of the strong ligaments at the base of the first metacarpal, resulting in the fracture-subluxation pattern we have here. Overall, the combination of intra-articular base of metacarpal fracture with subluxation (with or without OA) may mimic the appearance of trapeziectomy, which to the best of our knowledge has not been previously described in the literature.

Possible explanation for absence of multiple fracture fragments with this pattern of injury on plain film so soon after the injury would be of delayed presentation of an even older injury before this presentation, giving time for some fragments to remodel. This in addition to concurrent degenerative change at the carpometacarpal joint with some subluxation may explain some of the appearance. The recent fall may have re-fractured or re-injured the base of his thumb drawing the patient’s attention to this site. However, the patient does not recall any prior injury.

With the differing appearances between the plain films and the cross-sectional imaging, we would advocate for a low threshold for cross sectional imaging in cases with atypical features as plain radiographs may be deceiving.

## Conclusions

In conclusion, plain radiographs are the first-line investigation of hand injuries but have their limitations. In this case, a CT was able to delineate the true nature of the injury more accurately and we would advocate for a low threshold for cross-sectional imaging if appearances on plain film are atypical as the radiological appearance of metacarpal injury with concurrent degenerative changes may be misleading. Additionally, the hand is a functional piece of anatomy - this patient had good function despite the radiological appearance. This serves as a reminder that the patient, and not the imaging should be treated, and non-operative management may be appropriate and have good outcomes even in radiographically severe injuries.
